# Dietary Micronutrient Intake in Long-Term Survivors of Pediatric Hematopoietic Stem Cell Transplantation

**DOI:** 10.3390/nu17101663

**Published:** 2025-05-13

**Authors:** Louise Lindkvist Pedersen, Maria Ebbesen Sørum, Anne Nissen, Tina Gerbek, Karin Kok, Kaspar Sørensen, Martin Kaj Fridh, Christian Mølgaard, Klaus Gottlob Müller

**Affiliations:** 1 Department of Pediatrics and Adolescent Medicine, Copenhagen University Hospital—Rigshospitalet, 2100 Copenhagen, Denmark; louise.lindkvist.pedersen@regionh.dk (L.L.P.); maria.ebbesen.soerum@regionh.dk (M.E.S.); karin.kok@regionh.dk (K.K.); kaspar.soerensen.01@regionh.dk (K.S.); martin.kaj.fridh@regionh.dk (M.K.F.); 2 Pediatric Nutrition Unit, Copenhagen University Hospital—Rigshospitalet, 2100 Copenhagen, Denmark; cm@nexs.ku.dk; 3 Centre for Cancer and Organ Diseases, Copenhagen University Hospital—Rigshospitalet, 2100 Copenhagen, Denmark; 4 Department of Nutrition, Exercise and Sports, University of Copenhagen, 1172 Copenhagen, Denmark; 5 Institute of Clinical Medicine, Faculty of Health and Medical Sciences, University of Copenhagen, 2200 Copenhagen, Denmark; 6 Institute for Inflammation Research, Copenhagen University Hospital—Rigshospitalet, 2100 Copenhagen, Denmark

**Keywords:** pediatric hematopoietic stem cell transplantation, physical performance, body composition, late effects, nutrition, micronutrients

## Abstract

Background: Survivors of hematopoietic stem cell transplantation (HSCT) during childhood face significant late effects. This study aimed to map the dietary micronutrient intake of long-term survivors of pediatric HSCT and explore its associations with transplant outcomes, body composition, and physical capacity. Methods: We included 85 long-term survivors of HSCT (median age 30 years) The median time since HSCT was 19.9 years, reflecting a long-term survivor population. Dietary intake was assessed using a 3-day food record. Body composition was measured by DXA, and physical capacity was evaluated through cardiorespiratory fitness and physical performance tests. Results: We observed an inadequate intake of several vitamins and minerals including vitamins A, C, D, E, selenium, and potassium, with a median intake below recommendations. While dietary intake of vitamin D was reduced in patients with chronic graft versus host disease (cGvHD), the occurrence of cGvHD was not associated with overall micronutrient intake. Twelve percent of the participants had reduced skeletal muscle mass and 16% displayed a low bone mass density during DXA scans. These conditions were not related to the micronutrient intake. Likewise, reduced cardiorespiratory fitness and physical performance were unrelated to micronutrient intake. Total energy intake was found to significantly influence micronutrient intake (*p* = 0.001), explaining 66% of the variation. Conclusions: Long-term survivors of pediatric HSCT demonstrated inadequate intake of multiple micronutrients. These findings suggest that inclusion of comprehensive micronutrient assessment and nutritional guidance should be considered for inclusion in follow-up care protocols.

## 1. Introduction

Allogeneic hematopoietic stem cell transplantation (HSCT) during childhood is associated with significant late effects observed decades after the treatment [1,2,3] that are caused by the cytotoxic conditioning regimen as well as other treatment-related complications. Importantly, the conditioning treatment prior to HSCT includes chemotherapy, and in some cases, total body irradiation (TBI), which causes damaging effects on the gastrointestinal tract, with nausea, vomiting, diarrhea, and loss of appetite leading to impaired nutritional intake and malabsorption [4,5,6,7,8]. This has led to a concern that these early changes may lead to chronic malabsorption and persistent nutritional deficiency, highlighting the role of a sufficient dietary intake [4,5,6,7,8,9].

Common late effects observed in survivors of HSCT include cardiovascular disease, metabolic syndrome, osteoporosis, and sarcopenia [10,11,12,13], with an overall phenotype resembling the process of accelerated ageing being observed in the elderly population, but occurring decades earlier in HSCT survivors [2,14]. As the incidence and severity of these health conditions may be mitigated through lifestyle modifications in the background population, such as improved diet and increased physical activity [15], understanding survivors’ dietary patterns may be key in identifying opportunities to improve health outcomes through targeted dietary strategies.

While we and others have previously explored the relationship between macronutrient intake and late HSCT effects [16,17], insights into micronutrient intake in this population remains limited. The aim of this study was to provide a detailed characterization of the dietary micronutrient intake of long-term survivors of pediatric HSCT and to explore its associations with late effects, including body composition, cardiorespiratory fitness, and physical performance.

## 2. Materials and Methods

### 2.1. Study Design and Population

This study was part of a larger cross-sectional follow-up study of 96 long-term survivors of pediatric myeloablative allogeneic HSCT in Denmark. Eligibility criteria included undergoing HSCT before the age of 18 years between January 1980 and January 2018; being above 18 years old at the time of inclusion; and having completed a dietary registration. Exclusion criteria comprised any physical or mental illnesses that hindered participation, as well as pregnancy. Data from the same study population regarding the presence of metabolic syndrome, cardiorespiratory fitness, and physical performance have been published previously [10,18].

**Figure 1:**
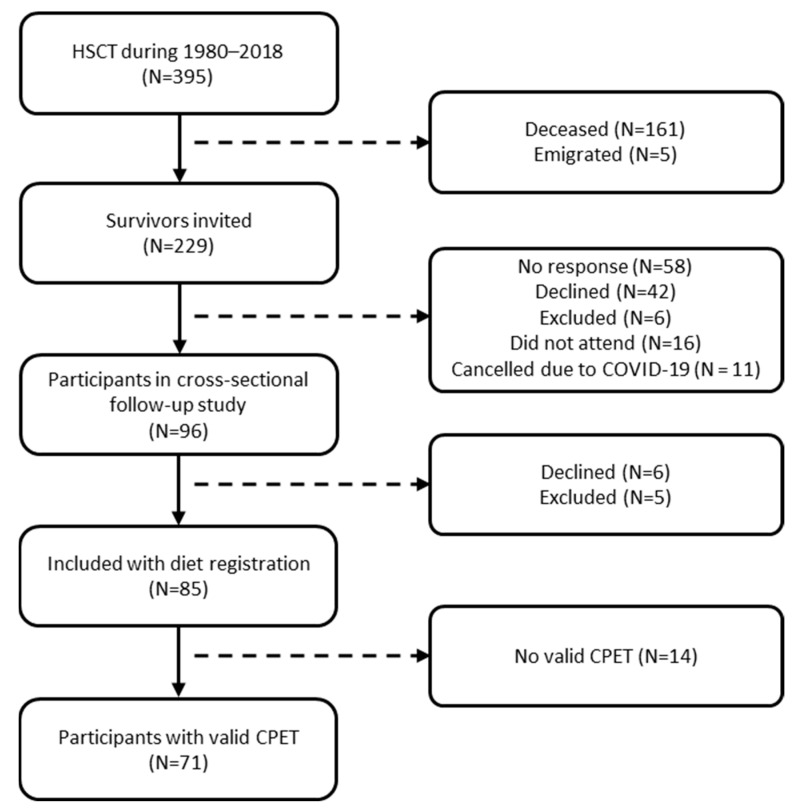
Flowchart for participants included in present study.

Out of the 96 eligible survivors, 11 either declined to participate or were unable to complete the dietary registration. These individuals will be referred to as non-participants. A total of 85 pediatric HSCT survivors completed the 3-day food record, comprising 44 males and 41 females. Of these, 71 performed a valid CPET (Figure 1).

### 2.2. Transplantation Variables

Information on diagnosis, conditioning regimens, donor matching, acute graft versus host disease (aGvHD), and other transplant-related variables were obtained from patient records. Donor matching was classified into three groups—HLA-matched sibling donors, matched unrelated donors, and other donors, including related and unrelated donors with one or more HLA mismatches.

Classification of aGvHD was performed according to the Glucksberg criteria [19,20] and the results were grouped into aGvHD grades 0-I and II-IV for analysis.

Chronic graft versus host disease (cGvHD) was assessed at the follow-up examination according to the National Institute of Health’s consensus criteria [21].

### 2.3. Dietary Record/Nutrition Intake Surveys

The intake of micronutrients and total energy was assessed using a 3-day food record. Participants were instructed to document all food and beverages consumed over two weekdays and one weekend day via the web-based software MADLOG [22]. Micronutrient contents for specific branded foods were retrieved from the manufacturers’ nutritional product labels, and for generic foods, they were retrieved from the national food composition database, Frida [23]. Missing or unclear data were clarified by participants. If any meals of the day were lacking, the reported amount seemed unlikely, or any other uncertainties were present, clarification was obtained through dialog with the participant.

The dietary intake of micronutrients was evaluated according to the Nordic Nutrition Recommendations (NNR 2023) [1] and the latest national dietary survey available for the general population (age 18–54 years) [24].

Recommended Intake (RI) [25] defines the daily dietary nutrient intake level that is sufficient to meet the nutrient requirements for nearly all (usually 97.5%) individuals in a particular life stage group in the general population, and is used as a guide for daily intake. For some nutrients (vitamins A, E, K, B12, phosphorus, potassium, magnesium, iodine, selenium), the RI is not defined due to a lack of appropriate data; in this case, Adequate Intake (AI) [25] recommendations are used for reference. In this study we will refer to both RI and AI collectively as “recommendations” as they both serve as reference values for specific micronutrients. During the illustration of micronutrient intake, we also applied Average Requirement (AR), which is the average daily nutrient intake level estimated to meet the requirements of half of the individuals in a particular life stage group in the general population [25]. For sodium specifically, a Chronic Disease Risk Reduction Intake (CDRR) is defined, representing the level of intake for which any reduction in sodium is expected to reduce the risk of chronic disease [25].

The included micronutrients comprised the following vitamins: A, D, E, B1, B2, niacin, B6, folate, B12, and C. The assessed minerals were calcium, phosphorus, magnesium, iron, zinc, iodine, selenium, sodium, and potassium. Based on the completed food records, the average daily intake of each micronutrient was calculated for each participant. Contributions from dietary supplements were calculated separately. Due to limitations in food composition databases and incomplete information on certain food items, not all micronutrients with a level recommended by the NNR were included in this analysis (pantothenic acid, biotin, choline, copper, fluoride, manganese, and molybdenum).

The energy intake was evaluated and classified according to reference levels in 2023’s NNR: sufficient (>8000 kJ/day), low (6500–8000 kJ/day), and very low (<6500 kJ/day). An intake below 6500 kJ per day carries a significant risk of micronutrient deficiencies [25].

### 2.4. Cardiorespiratory Fitness Assessment

Participants underwent a cardiopulmonary exercise test (CPET) on an electronically braked cycle ergometer (Lode Corival Pediatric or Monark Ergomedic 839 E) using a modified Godfrey protocol [26]. A breath-by-breath system (INNOCOR ergo-spirometry-system, Innovision, DK-5260 Odense, Denmark) measured the maximum oxygen uptake (VO2 peak), defined as the highest mean oxygen uptake over 60 s, expressed as absolute values (L/min) and relative values (mL/kg/min). The peak workload was recorded as the maximal watts produced. Heart rate and oxygen saturation were monitored every 30 s (Polar FT2 sport tester, Polar Electro, Kemple, Finland).

To validate CPETs, the following three criteria were used: (i) subjective assessment of intense effort, (ii) heart rate > 180 bpm, and (iii) respiratory exchange rate ≥ 1.1. The test was halted if oxygen saturation dropped below 90% or if participants could not maintain a minimum cadence of 70 rpm.

### 2.5. Physical Performance Tests

Handgrip Strength (right and left): Handgrip strength was measured with participants standing with elbows flexed at 90 degrees, free from the body, using a Saehan hand dynamometer (Glanford Electronics, Scunthorpe, UK) [27]. Each arm was tested twice, with the highest value (kg) recorded for analysis.

Timed-Up-and-Go (TUG): Participants started seated, then stood, walked 3 m, turned, returned, and sat down as quickly as possible [28]. The time (s) of the fastest of three attempts was used for analysis.

Sit-to-Stand (30 and 60 s): Participants sat on a chair, then stood up with knees and hip extended, and sat down again as many times as possible within 60 s. The number of repetitions was recorded at 30 and 60 s [29].

6-Minute Walk Test: Participants walked as far as possible in 6 min on a 15 m straight indoor track. The total distance (m) was measured [30].

Walking Pace: Participants walked a 15 m straight indoor track as fast as possible, with the fastest pace (m/s) of three attempts recorded for analysis [31].

### 2.6. Body Composition

The total body Bone Mineral Density (BMD) z-score [32] was assessed using Dual-Energy X-ray Absorptiometry (DXA) with a Lunar Prodigy Advance fan beam scanner (GE Medical Systems, Madison, WI, USA) using Prodigy enCORE software version 16.10.151. Skeletal muscle mass was calculated from DXA-derived lean mass, expressed as Skeletal Muscle Index (SMI) determined by dividing appendicular skeletal muscle mass (ASM) by height squared (m^2^): SMI = ASM/height^2^. The SMI z-score was calculated using the following formula: Z-score = (SMI participant − SMI mean of reference group)/SMI standard deviation of reference group [33], allowing for comparison against age- and sex-matched normative data.

### 2.7. Plasma Analysis

Laboratory analysis included the measurement of plasma 25-hydroxyvitamin D (25(OH)D) (nmol/L) and plasma ferritin concentration. Both 25(OH)D and ferritin (ng/mL) measurements were performed on fasting blood samples collected from participants. All analyses were performed at the Department of Clinical Biochemistry, Copenhagen University Hospital, Rigshospitalet.

### 2.8. Statistics

Data are presented as the median and range unless otherwise specified.

To assess differences between participants and non-participants regarding demographic and clinical characteristics, the Mann–Whitney U-test was used to compare age at the time of the examination, time from HSCT, and age during HSCT. Fisher’s exact test was used to compare diagnoses, conditioning regimens, donor matches, and prevalence of aGvHD. The Mann–Whitney U-test was used to assess associations between continuous variables and cGvHD. Spearman’s rank order correlation coefficient was used to evaluate correlations between micronutrients and body composition and cardiorespiratory fitness.

Multiple regression analysis was performed to examine the relationship between micronutrients and physical performance, adjusting for age and sex, as both factors are known to influence physical outcomes [34].

To explore the multivariate relationships among samples with regard to micronutrient intake in relation to energy intake, Principal Coordinate Analysis (PCoA) was performed using a distance matrix derived from the Euclidean method [35]. Differences in micronutrient composition among groups were tested using permutational multivariate analysis of variance (PERMANOVA) (adonis2 with 999 permutations).

Multiple test correction was performed using the Benjamini–Hochberg False Discovery Rate (FDR) method for comparisons between micronutrients and body composition as well as physical tests, with an FDR threshold of 0.05.

In all statistical analyses a *p*-value of less than 0.05 was deemed statistically significant. Statistical analyses were carried out using R (R Foundation for Statistical Computing, Vienna, Austria), version 4.1.0 [36].

### 2.9. Ethics

The study was approved by the Regional Committee on Health Research Ethics (H-18020790) and by the Danish Data Protection Agency (2018-271-6511) and conducted in accordance with the Declaration of Helsinki. All participants gave written and oral informed consent before enrolling in the study.

## 3. Results

A total of 85 pediatric HSCT survivors were included in this study. The median follow-up time since HSCT was 19.9 years (range: 5.9–36.9 years), reflecting the long-term nature of this survivor cohort. The median BMI in our cohort was 23.1 kg/m^2^ (range: 15.6–31.9), which is lower than the median BMI reported for the general Danish population (reference value: 26.1 kg/m^2^). The underlying diagnoses among the study cohort were as follows: acute leukemia in 42 patients (49%), other malignant diseases in 15 patients (18%), and benign diseases in 28 patients (33%).

HSCT, hematopoietic stem cell transplantation; CPET, cardiopulmonary exercise test.

No significant differences were found between participants’ and non-participants’ characteristics in terms of age at the time of the study, time from HSCT, diagnosis, age during HSCT, conditioning regimen, donor match, or prevalence of aGvHD or cGvHD. 

The characteristics of the survivors are summarized in Table 1.

### 3.1. Vitamins

**Figure 2:**
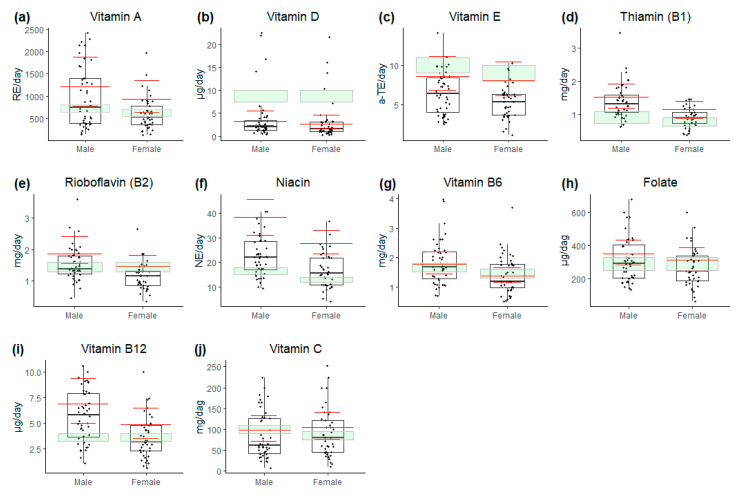
Daily dietary intake of vitamins. (**a**) Vitamin A (RE/day); (**b**) vitamin D (µg/day); (**c**) vitamin E (α-TE/day); (**d**) thiamin (mg/day); (**e**) riboflavin (mg/day); (**f**) niacin (NE/day); (**g**) vitamin B6 (mg/day); (**h**) folate (µg/day); (**i**) vitamin B12 (µg/day); (**j**) vitamin C (mg/day). Data are presented as median values with interquartile range. Study population is represented by white boxplots, while median and interquartile range for general population are indicated by red lines. Green shaded areas (RI/AI as upper boundary and AR as lower boundary) indicate recommendations according to Nordic Nutrition Recommendations 2023 [25].

For the median dietary intake of vitamins (Figure 2), only thiamin (B1) and niacin met the recommendations for both females and males, while the median intake of vitamins A, C, D, E, B2, B6, and folate all fell below the recommended levels. Only females had a vitamin B12 intake level below the recommended level. The intake of vitamins E and D was particularly low, with 96% (82/85) having a vitamin E intake below the recommended level, and a total of 91% (77/85) had a vitamin D intake below the recommended level.

In comparison with the background population (Figure 2), the study group generally demonstrated lower median intakes, with the most pronounced differences observed for niacin, vitamin A, and vitamin E, while vitamin D intake in the background population was comparably low.

### 3.2. Vitamin D Plasma Levels and cGvHD

**Figure 3:**
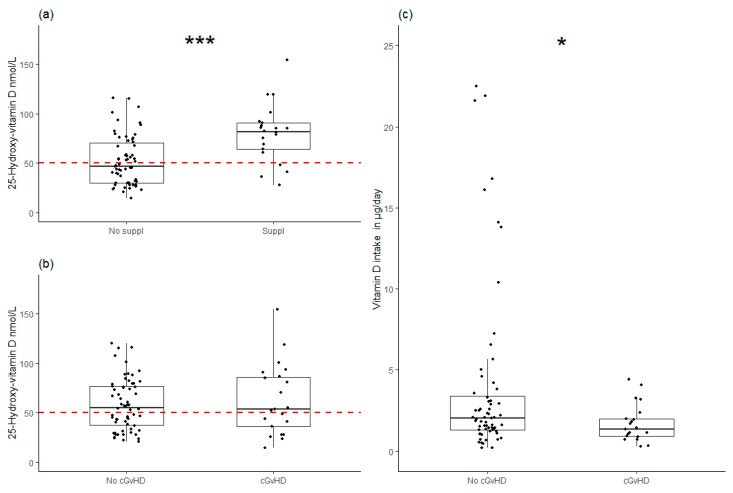
Associations between vitamin D and chronic graft versus host disease (cGvHD). (**a**) Vitamin D plasma levels in relation to vitamin D supplement intake (*** *p* = 0.00018, statistics: Mann–Whitney). (**b**) Vitamin D plasma levels in relation to cGvHD. (**c**) Daily dietary intake of vitamin D in relation to cGvHD (* *p* = 0.049, statistics: Mann–Whitney). Dashed red lines represent minimum recommended concentration of 25-hydroxyvitamin D.

We found that 43% (37/85) of the participants had a vitamin D plasma level below 50 nmol/L, which is the minimum recommended concentration of 25(OH)D. We further addressed the use of vitamin D supplements and plasma levels of vitamin D in addition to the overall food-related vitamin D intake. A total of 21 participants (32%) reported taking a daily supplement of vitamin D, and of these, 16 (76%) reported using a combined calcium and vitamin D supplement. We observed a significantly higher vitamin D plasma level among these individuals (82 nmol/L [28–154 nmol/L] vs. 47 nmol/L [15–116 nmol/L], *p* = 0.00018) (Figure 3a).

In participants with cGvHD, dietary vitamin D intake was reduced compared to participants without cGvHD (1.4 µg/day [0.3–4.4 µg/day] vs. 2.1 µg/day [0.2–22.5 µg/day], *p* = 0.049) (Figure 3c), and plasma levels were reduced in these participants to a similar degree to that seen in participants without cGvHD (54 nmol/L [15–154 nmol/L] vs. 55 nmol/L [21–120 nmol/L], *p* = NS) (Figure 3b). Less than one third of participants with cGvHD were taking a daily vitamin D supplement (9/21, 32%). cGvHD was unrelated to the other micronutrients investigated in this study.

### 3.3. Minerals

**Figure 4:**
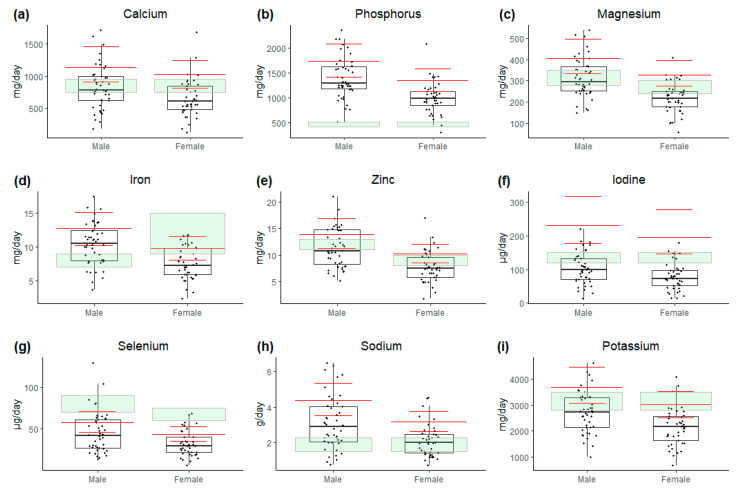
Daily dietary intake of minerals. (**a**) Calcium (mg/day); (**b**) phosphorus (mg/day); (**c**) magnesium (mg/day); (**d**) iron (mg/day); (**e**) zinc (mg/day); (**f**) iodine (µg/day); (**g**) selenium (µg/day); (**h**) sodium (g/day); (**i**) potassium (mg/day). Data are presented as median values with interquartile range. Study population is represented by white boxplots, while median and interquartile range for general population are indicated by red lines. Green shaded areas (RI/AI as upper boundary and AR as lower boundary) indicate recommendations according to Nordic Nutrition Recommendations 2023 [25].

Regarding the median dietary intake of minerals (Figure 4), only phosphorus met recommendations in both females and males (83/85). The median dietary intake of iron only met recommendations in males (29/44). Given the risk of iron overload due to previous multiple blood transfusions, we also assessed ferritin levels. We found that 10% of the study population (8 males and 1 female) had a ferritin level exceeding 500 ng/mL, with 4 individuals having a mild iron overload (500–999 ng/mL) and 5 having a moderate iron overload (1000–1999 ng/mL) [37]. No associations between ferritin levels and iron intake, diagnosis, or time to follow-up were found. The median dietary intake of zinc, iodine, selenium, and potassium fell below the recommended levels for both females and males. Additionally, calcium and magnesium intake fell below the recommended levels for females.

Sodium intake was above the CDRR in 49% (42/85) of the study population, corresponding to 2.3 g/day (5.75 g of salt pr day).

In comparison with the background population (Figure 4), the study population generally demonstrated a lower median intake of all minerals.

### 3.4. Body Composition

The SMI z-scores among participants had a median of −1.0 (−3.9 to 1.9). Notably, 12% (10/82) of participants had z-scores below −2, indicating a reduced skeletal muscle mass, with one individual scoring below −3. The median BMD z-score was 0.4 (−3.6 to 4.0). Among the participants, 15.5% (13/83) had a z-score < −1, which indicates a reduced BMD. One individual had a z-score < −2, which suggests a very low BMD. No significant associations were found between dietary intake of micronutrients and either SMI z-score or BMD z-score.

### 3.5. Cardiorespiratory Fitness and Physical Performance

We explored associations between cardiorespiratory fitness, physical performance, and micronutrient intake. In comparison with age-matched controls, a large proportion of the participants manifested with reductions in their cardiorespiratory fitness level and physical performance test results, as reported previously [38]. Reduced cardiorespiratory fitness and physical performance were unrelated to the micronutrient intake.

### 3.6. Micronutrients in Relation to Energy Intake

The median intake of energy among the participants was 7656 kJ/day (6464–9704 kJ/day). The median energy intake for males was 9330 kJ/day (7760–10807 kJ/day), and for females, 6624 kJ/day (6181–7467 kJ/day).

Notably, more than half of the study population (49/85, 15 males and 34 females) reported an energy intake below 8000 kJ, and 25% (22/85, 5 males and 17 females) had an intake below 6500 kJ.

**Figure 5:**
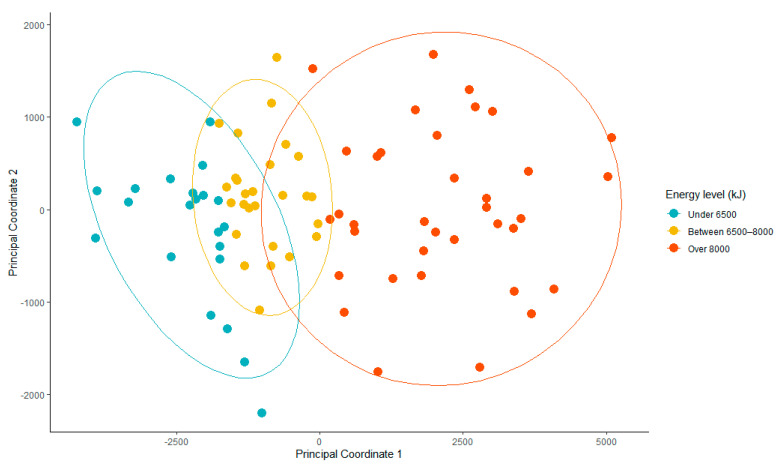
Principal coordinate analysis of micronutrient intake in relation to energy intake. Plot shows principal coordinates (PC1 and PC2), which together explain largest proportion of variation in micronutrient data. Points represent individual participants. Differences in colors indicate energy level categories (normal, low, very low). PC1 accounts for largest proportion of variance and is strongly correlated with energy intake (loading = 0.99). PC2 represents second largest source of variation, capturing patterns in micronutrient intake that are independent of total energy consumption. Ellipses represent 95% confidence intervals for each energy level category. PERMANOVA analysis confirmed significant effect of energy intake categories on micronutrient profiles (*p* = 0.001), with energy intake categories explaining 65.5% of total data variation.

Exploring associations between patterns in micronutrient intake and energy intake, we found a significant effect of energy intake on micronutrient profiles (F = 77.9, *p* = 0.001), with energy intake categories explaining 65.5% of the total variation in micronutrient profiles (Figure 5).

PCoA was conducted to visualize and explore the patterns in micronutrient intake. Analysis of the loadings on PC1 revealed that energy intake had the strongest correlation with PC1 (loading = 0.99). The micronutrients that contributed most strongly to PC1 were phosphate, zinc, magnesium, iron, and vitamin B2, suggesting that higher energy intake is associated with increased consumption of these micronutrients.

## 4. Discussion

We studied the dietary intake of micronutrients among pediatric survivors of HSCT in relation to the official recommendations [25]. The intake of the majority of micronutrients, vitamin A, C, D, E, B2, B6, and folate, as well as the minerals zinc, iodine, selenium, and potassium were found to be below the current recommendations for the healthy background population, aligning with two previous studies [39,40]. A study of adult HSCT survivors found that 80% had a poor quality diet with a specifically inadequate intake of vitamins A, C, D, folate, magnesium, and calcium [39]. A large study by Zhang et al. [40] examined the dietary habits of 2570 adult survivors of childhood cancer enrolled in the St. Jude Lifetime cohort and found that survivors had poor adherence to the 2010 Dietary Guidelines for Americans. Specifically, survivors consumed inadequate amounts of vitamin D, vitamin E, potassium, magnesium, and calcium, while they consumed excessive amounts of sodium [40].

We examined the relationship between cGvHD and micronutrient intake and found that participants with cGvHD had a lower dietary intake of vitamin D. Vitamin D plays a crucial role in calcium and bone homeostasis, in addition to various other physiologic functions, including immune functions, and is a potent inhibitor of inflammatory cytokine production [41]. Deficiencies have been linked to increased risk of infections, autoimmune diseases, and complications following HSCT [2,3,42,43]. Therefore, our findings of insufficient vitamin D plasma levels in almost half of the survivors is of potential concern and in concordance with other studies [44,45]. Of note, the severity of cGvHD has been associated with vitamin D deficiency, suggesting a potential protective effect of vitamin D supplementation [46,47]. Therefore, it is noteworthy that less than one third of the patients with cGvHD were taking vitamin D supplements, raising concerns about the overall effectiveness of the current supplementation strategies and patient guidelines.

Iron status has a specific focus in post-transplant follow-up as many of the survivors exhibit iron overload in the early years post-transplant due to multiple blood transfusions given during the phase of aplasia in the immediate period after HSCT [37]. For younger women in the background population, dietary iron content often fails to meet the recommended intake [1], which was also found in our study population. An increased intake of iron-rich foods may be necessary in the background population. Due to the risk of iron overload after HSCT, caused by multiple blood transfusions, it is essential to assess ferritin plasma levels to determine whether increased iron intake is safe in long-term survivors of HSCT.

We observed high sodium consumption, primarily from prepared foods and meals. This may be of concern due to potential health implications hereof including increased risks of stroke, cardiovascular events, and mortality in the general adult population [48]. Both for the study population and for the background population, the intake is based on salt added industrially or as part of a recipe, while salt added at the table is not determined. Accordingly, the actual total salt intake is likely to be even higher. Given the already increased risk of cardiovascular disease in HSCT survivors [49], these findings emphasize the importance of addressing the sodium intake in this vulnerable population.

Studies in the general population have reported associations between general health conditions and micronutrient intake [50,51,52]. Although our study highlighted the insufficient intake of these micronutrients, which could potentially contribute to low SMI and BMD, we were not able to show significant associations between micronutrient intake and altered body composition or reduced cardiorespiratory fitness and physical performance in the HSCT survivors. Of note, the vitamins subjected to reduced intake included vitamin D as well as calcium, magnesium, and zinc, which are known to play essential roles in bone metabolism and muscle function [25,53]. The lack of significant associations in our study may be due to differences in study populations where the toxicity of the HSCT procedure plays a relatively larger role on these outcomes than the nutrition as such. Importantly, due to our relatively small sample size, the absence of significant correlations should be taken with caution. Furthermore, factors such as age, physical activity level, and overall health status may influence these associations, as they are well-known to influence body composition and physical capacity [47,48,49].

Evidence suggests that dietary patterns play a crucial role in shaping the composition and function of the gut microbiota, which in turn can influence immune regulation and long-term health outcomes [54], particularly in populations with altered immune systems such as HSCT survivors. The gut microbiota is known to be affected by the diet, which can modulate microbial diversity and metabolic activity. In HSCT recipients, disruptions of the gut microbiome have been associated with complications such as GvHD and increased susceptibility to infections [55,56]. Although our study did not directly assess gut microbiota composition, the observed inadequacies in micronutrients may have downstream effects on microbiome health and, consequently, on immune function and inflammation. These relationships warrant further investigation in future studies, ideally incorporating both dietary assessment and direct analysis of the gut microbiome to better understand the interplay between nutrition, microbiota, and long-term outcomes after HSCT.

The finding of insufficiencies in the intake of several micronutrients raises questions regarding the reasons for this. We found that a large proportion of the survivors had a relatively low energy intake, with this being most pronounced among females, and importantly, our results revealed distinct associations between energy intake and the intake of several micronutrients. We found that a greater intake of phosphate, zinc, iron, magnesium, and vitamin B2 was associated with higher energy intake, likely reflecting the fact that energy-dense foods like dairy, starchy vegetables, meats, and processed items are rich in such micronutrients. However, while such diets meet certain micronutrient requirements, they also carry a risk of excess caloric intake, which emphasizes the need to balance energy and micronutrient density in dietary recommendations. These considerations are of particular importance in childhood cancer survivors due to their increased risk of metabolic syndrome [3,4].

While our study has several strengths, including a long follow-up period (median 20 years) and the use of self-reported dietary records, it has some limitations. The extended median time since HSCT reflects a long-term survivor cohort, which is important to consider when interpreting the findings. Over such a prolonged interval, multiple factors beyond the transplant procedure itself—such as aging, lifestyle changes, and other health conditions—may influence current dietary patterns and nutritional status. Also, while early post-transplant malabsorption is well documented due to mucosal injury and graft versus host disease, there is a lack of data on micronutrient absorption decades after HSCT. Our study did not assess absorption capacity, and future research should address whether long-term survivors experience persistent alterations in nutrient uptake. With regard to the dietary patterns, these were self-reported rather than directly measured, which may have led to underreporting of caloric intake [48,49]. Previous studies have identified a tendency for selective underreporting, particularly for unhealthy components such as fat and sugar, while healthier items such as vegetables tend to be overreported [57]. Accordingly, the findings in the present study of suboptimal intake of dietary micronutrients may very well be understated. Furthermore, we did not collect data on participants’ intentions to lose or gain weight. Since such intentions can influence dietary behavior, this represents an additional limitation that should be addressed in future studies.

Importantly, we measured plasma concentrations of vitamin D and ferritin, which enabled us to directly assess the status of these specific micronutrients. However, for other micronutrients such as vitamins A, C, E, and selenium, only dietary intake was evaluated. While dietary data provide valuable insights, a broader range of biochemical assessments would offer a more comprehensive evaluation of nutritional status and should be considered in future studies. It is also important to note that plasma levels of micronutrients do not always directly reflect recent dietary intake, as circulating concentrations can be influenced by factors such as absorption efficiency, metabolic processes, tissue storage, inflammation, and underlying health conditions that may be altered in survivors of HSCT, further stressing the relevance plasma levels of micronutrients.

The observational nature of the study, the sample size, and the cohort heterogeneity may limit the generalizability of our findings. Furthermore, the risk of selection bias, with the preferential recruitment of survivors with a relatively healthy lifestyle, could result in an underestimation of reduced physical capability and dietary intake issues.

Given the similarities between the late effects experienced by HSCT survivors and the lifestyle-related diseases in the background population, it is plausible that lifestyle interventions, including dietary intervention, could be beneficial for HSCT survivors, and this warrants further investigation.

Overall, this study provides valuable insight into the dietary intake of micronutrients in survivors of pediatric HSCT, as the role of micronutrients in overall health and recovery post-HSCT may be critical. Our findings stress the need for further investigations into this to further define the role of micronutrient assessment and supplementation in follow-up care protocols for survivors of HSCT.

Future research should focus on longitudinal studies that further explore the micronutrient status of HSCT survivors, particularly regarding potential long-term effects of deficiencies on health outcomes. Intervention studies and randomized controlled trials assessing the impact of dietary guidance and/or micronutrient supplementation on recovery and quality of life in this population would provide valuable insights into effective management strategies.

## 5. Conclusions

Long-term survivors of pediatric hematopoietic stem cell transplantation (HSCT) in this study demonstrated a consistently inadequate dietary intake of several key micronutrients, including vitamins A, C, D, E, selenium, and potassium, with most participants failing to meet established nutritional recommendations. Despite these deficiencies, no significant associations were observed between micronutrient intake and measures of body composition, bone mineral density, skeletal muscle mass, or physical performance. Notably, total energy intake emerged as the primary determinant of overall micronutrient intake, accounting for a substantial proportion of the observed variation. These findings underscore the importance of comprehensive nutritional assessment and targeted dietary guidance as integral components of long-term follow-up care for pediatric HSCT survivors, with the aim of mitigating potential late effects and supporting optimal health outcomes.

## Figures and Tables

**Table 1:** Survivor and transplantation characteristics.

Characteristics	
Included survivors, n Males, n (%)	85 44 (52)
Patient-related characteristics, median (range)	
Age at follow-up, years	30.4 (19.6–53.0)
Weight, kg	63.0 (36.0–98.6)
BMI, kg/m^2^	23.1 (15.6–31.9)
Time from transplantation to examination, years	19.9 (5.9–36.9)
Age at transplantation, years	10.9 (0.4–17.9)
Donor age, years	11.5 (1.7–47.6)
Diagnosis, n (%)	
Acute leukemia	42 (49)
Other malignant disease	15 (18)
Benign disease	28 (33)
Donor type, n (%)	
HLA-identical sibling	45 (53)
Matched unrelated donor	23 (27)
Others	17 (20)
Graft type, n (%)	
Bone marrow	78 (92)
Peripheral blood	2 (2)
Umbilical cord blood	5 (6)
Conditioning, n (%)	
TBI-based conditioning regimes	45 (53)
BU + CY	24 (28)
Other	16 (19)
GvHD, n (%)	
Acute GvHD	
Grade 0-I	60 (70)
Grade II-IV	25 (30)
Chronic GvHD	
Yes	21 (25)
No	64 (75)

Abbreviations: BMI: Body mass index; HLA: human leukocyte antigen; TBI: total body irradiation; BU: busulfan; CY: cyclophosphamide; GvHD: graft versus host disease. Acute GvHD was graded according to Glucksberg criteria [19,21].

## Data Availability

The data that support the findings of this study are available from the corresponding author upon reasonable request.

## Abbreviations

**Abbreviation**	**Full term**
HSCT	Hematopoietic stem cell transplantation
TBI	Total body irradiation
aGvHD	Acute graft versus host disease
HLA	Human leukocyte antigen
cGvHD	Chronic graft versus host disease
RI	Recommended intake
AI	Adequate intake
AR	Average Requirement
CPET	Cardiopulmonary exercise test
VO2 peak	Maximal oxygen uptake
BMD	Bone Mineral Density
DXA	Dual-Energy X-ray Absorptiometry
SMI	Skeletal Muscle Index
ASM	Appendicular skeletal muscle mass
RE	Retinol equivalents
α-TE	Alpha-tocopherol equivalents
NE	Niacin equivalents
CI	Confidence interval
